# Identification of Salivary Microorganisms and Metabolites Associated with Halitosis

**DOI:** 10.3390/metabo11060362

**Published:** 2021-06-07

**Authors:** Jae-kwon Jo, Seung-Ho Seo, Seong-Eun Park, Hyun-Woo Kim, Eun-Ju Kim, Chang-Su Na, Kwang-Moon Cho, Sun-Jae Kwon, Young-Ho Moon, Hong-Seok Son

**Affiliations:** 1 Department of Biotechnology, College of Life Sciences and Biotechnology, Korea University, Seoul 02841, Korea; jojk89@naver.com (J.-k.J.); parkse@korea.ac.kr (S.-E.P.); mn40120@naver.com (H.-W.K.); 2 Sonlab Inc., Seoul 02841, Korea; blue784300@naver.com; 3 Department of Korean Medicine, Dongshin University, Naju 58245, Korea; yci3431@naver.com (E.-J.K.); csna@dsu.ac.kr (C.-S.N.); 4 AccuGene Inc., Incheon 22006, Korea; kmcho@accugenelab.co.kr (K.-M.C.); sjaes@accugenelab.co.kr (S.-J.K.); 5 Naju Korean Medical Hospital, Dongshin University, Naju 58326, Korea

**Keywords:** halitosis, microbiome, metabolomics, cadaverine, putrescine

## Abstract

Halitosis is mainly caused by the action of oral microbes. The purpose of this study was to investigate the differences in salivary microbes and metabolites between subjects with and without halitosis. Of the 52 participants, 22 were classified into the halitosis group by the volatile sulfur compound analysis on breath samples. The 16S rRNA gene amplicon sequencing and metabolomics approaches were used to investigate the difference in microbes and metabolites in saliva of the control and halitosis groups. The profiles of microbiota and metabolites were relatively different between the halitosis and control groups. The relative abundances of *Prevotella*, *Alloprevotella*, and *Megasphaera* were significantly higher in the halitosis group. In contrast, the relative abundances of *Streptococcus*, *Rothia*, and *Haemophilus* were considerably higher in the control group. The levels of 5-aminovaleric acid and n-acetylornithine were significantly higher in the halitosis group. The correlation between identified metabolites and microbiota reveals that *Alloprevotella* and *Prevotella* might be related to the cadaverine and putrescine pathways that cause halitosis. This study could provide insight into the mechanisms of halitosis.

## 1. Introduction

The term ‘halitosis’ is used to describe an unpleasant odor emanating from the mouth, regardless of the cause or origin of the malodor [1]. About 50% of the population worldwide view themselves as having halitosis, and 10–30% of the population need ongoing care or treatment [2]. Halitosis is largely divided into transient halitosis, extra-oral halitosis (EOH), and intra-oral halitosis (IOH) [3]. EOH is divided into bloodborne (diabetes, kidney, and liver disease) and non-bloodborne (respiratory and gastrointestinal diseases), accounting for 5–10% of total halitosis [3,4]. IOH, which accounts for 80–90% of total halitosis cases [3], is related to oral conditions, such as tongue coating, gingival and periodontal disease, deep carious lesions, and peri-implant diseases [5]. IOH is mainly caused by the putrefactive actions of microorganisms such as bacteria, fungi, viruses, and protozoa [6].

After the distal intestine, the oral cavity has the second most diverse microbial population in the human body [4]. Pathological conditions in the oral cavity are responsible for 80–90% of IOH [7]. Some studies showed that the bacterial composition and diversity of the IOH group are different from those of the control group [8,9]. Previous studies have reported that bacteria to produce of volatile sulfur compounds (VSCs), such as *Solobacterium moorei*, *Prophyromonas gingivalis*, *Treponema denticola*, *Prevotella intermedia*, *Streptococcus oralis*, and *Tannerella forsythia*, are associated with IOH [10,11,12,13].

The main cause of IOH is VSCs produced by oral bacteria. In particular, hydrogen sulfide (H_2_S), methyl mercaptan (methanethiol, CH_3_SH), and dimethyl sulfide (C_2_H_6_S) are considered important markers of IOH [14]. As a method of measuring VSCs, VSC monitors such as the Halimeter are most commonly used [11,15]. However, this method has the disadvantage that the Halimeter does not distinguish between the different VSCs, giving only a total VSC measurement, and being a portable sulphide monitor [16]. Moreover, the presence of alcohols, phenyl compounds, and polyamines can interfere with the readings [17]. In addition, in the assessment of IOH using the Halimeter, substances other than VSCs, but presumed to cause bad breath, such as putrescine, cadaverine, indole, and skatole, which are not detectable by a sulfide monitor, are often not considered [18,19,20,21].

As part of a systems biology approach, metabolomics can improve our understanding of complex cellular pathways and biological mechanisms in halitosis [22]. Gas chromatography–mass spectrometry (GC–MS) provides an important means of generating metabolomics data, in which all metabolites that may cause IOH, including VSCs, in an untargeted method are profiled [22,23]. IOH is caused by secondary compounds released by microorganisms found in the oral cavity [24]. Therefore, understanding IOH depends on identifying the microbes that alter production of oral metabolites. Oral bacteria degrade sulfur-containing amino acids (cysteine, homocysteine, and methionine) to generate VSCs and various other metabolites [25,26,27]. Recent studies reported the relationship between the microbiome and metabolome in disease development such as nonalcoholic fatty liver disease, diabetes, insulin resistance, and obesity [28,29]. The present study aimed to use 16S rRNA amplicon sequencing and GC–MS-based metabolite profiling to elucidate the differences in microbiome and metabolite composition of the saliva from subjects with or without halitosis.

## 2. Results and Discussion

### 2.1. Demographic and Clinical Characteristics of the Study Subjects

Of the 52 participants, 22 subjects (13 females and 9 males) complied with the criteria for halitosis, whereas 30 subjects (20 females and 10 males) complied with the criteria for the control group. The mean ages of the control and halitosis groups were 38.50 ± 11.94 (range 20–63) years and 43.43 ± 15.73 (range 21–65) years, respectively. The demographic and clinical characteristics of the study population and are listed in Table 1.

### 2.2. Profiling of Salivary Bacterial Microbiome

**Figure 1:**
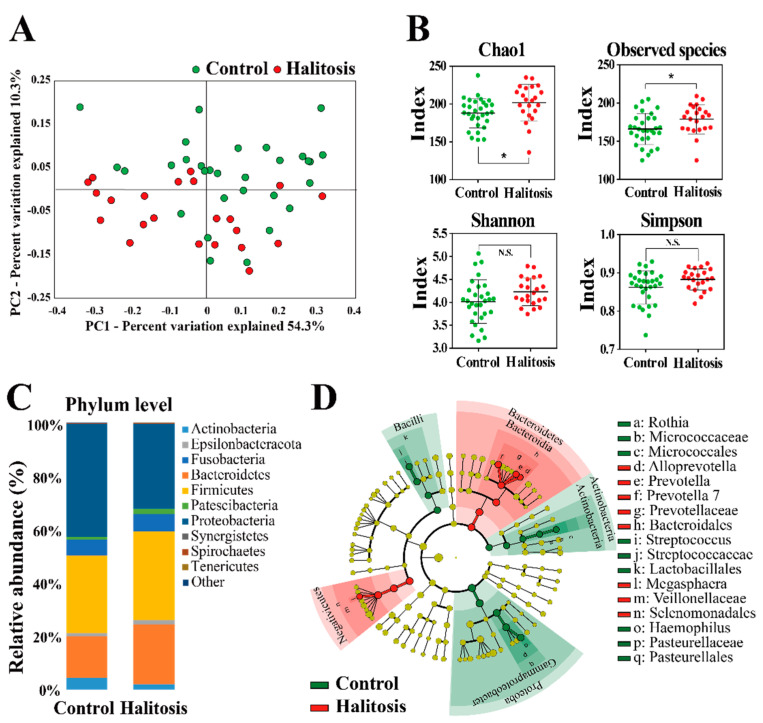
Comparison of the diversity and taxonomy of salivary microbiota according to halitosis. (**A**) The principal coordinates analysis (PCoA) based on the Bray-Curtis distances of salivary microbiota between the control and halitosis groups. (**B**) Comparison of the alpha diversity of salivary microbiota between the control and halitosis groups. (**C**) Comparison of the microbiota composition between the control and halitosis groups at the phylum level. 16S rRNA gene sequences were clustered into the operational taxonomic units (OTUs) based on 97% identity. OTUs with >1% relative abundance are represented in the phyla. (**D**) Cladogram showing the most discriminative bacterial clades identified using linear discriminant analysis effect size (LEfSe). Colored region/branches indicate differences in the bacterial population structure between the control group and the halitosis group. Sectors in green indicate clades that are enriched in the control group compared with the halitosis group, whereas sector in red indicates clades that are enriched in the halitosis group compared with the control group. * *p* < 0.05, N.S., Not Significant.

To evaluate the variability of microbial communities between the two groups, beta diversity was assessed using the principal coordinates analysis (PCoA) [30]. Based on Bray–Curtis dissimilarities, a distinct clustering was found in the PCoA plot between the control and halitosis groups, although some samples overlapped (Figure 1A). To estimate differences in microbial diversity among groups, alpha diversity (Chao1, Shannon, Simpson, and observed species indices) was analyzed. Kazor et al. [9] reported a significantly higher variety of oral bacteria in the halitosis group compared with the control group, suggesting that bacterial diversity might be a putative factor of halitosis.

In alpha diversity analysis, the Chao1 and observed species diversity indices were higher in the halitosis group than in the control group (Figure 1B). The Shannon and Simpson diversity indices between the control and the halitosis groups were not significantly different because of the high variability observed. Oshiro et al. [31] reported that the Shannon and Chao1 diversity indices were significantly higher in the halitosis group than in the non-halitosis group. Analysis of the bacterial composition at the phylum level revealed that Firmicutes, Fusobacteria, Actinobacteria, Bacteroidetes, Proteobacteria, Patescibacteria, and Epsilonbacteraeota were present in all saliva samples, regardless of the halitosis status (Figure 1C). Differentially abundant taxa were further verified using linear discriminant analysis effect size (LEfSe). LEfSe is an algorithm for the high-dimensional biomarker discovery that exploits linear discriminant analysis (LDA) to robustly identify statistically different features among classes [32]. Figure 1D shows the most relevant clades identified using LEfSe (LDA score > 3.0).

**Figure 2:**
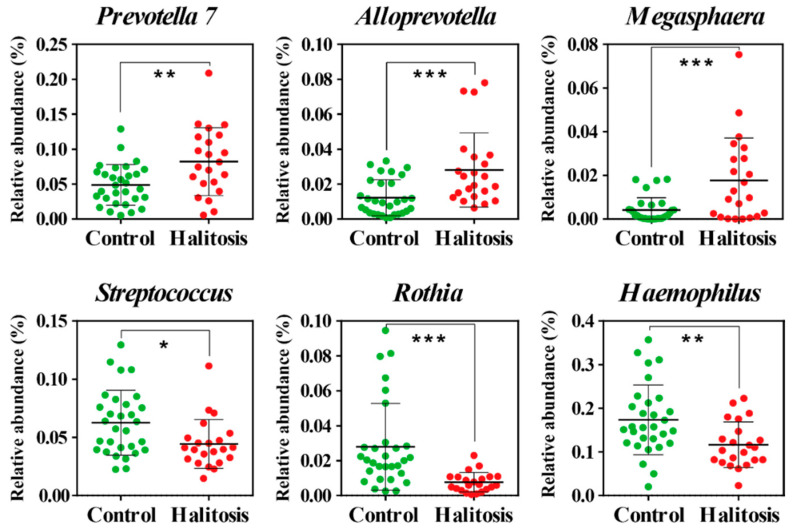
Scatter dot plots of bacteria genera identified by linear discriminant analysis effect size (LEfSe) (LDA score > 3.0) to be differentially abundant between the halitosis and control groups. *p*-values were obtained using Mann–Whitney U-tests. * *p* < 0.05; ** *p* < 0.01; *** *p* < 0.001.

Significant differences in the bacterial composition between the control and halitosis groups were found at the genus level. The relative abundances of *Prevotella* (*p* < 0.01), *Alloprevotella* (*p* < 0.001), and *Megasphaera* (*p* < 0.001) were significantly higher in the halitosis group than in the control group. The relative abundances of *Streptococcus* (*p* < 0.05), *Rothia* (*p* < 0.001), and *Haemophilus* (*p* < 0.01) were significantly higher in the control group (Figure 2) than in the halitosis group. The bacteria with a high relative abundance in the halitosis group may be specifically associated with the production of metabolites that cause halitosis. Ye et al. [33] reported that *Alloprevotella* was an important genus in halitosis, suggesting that *Alloprevotella* had a positive association with halitosis. Takeshita et al. [34] reported that samples collected from patients with halitosis showed a dominance of the genus *Megasphaera*. *Prevotella* is an abundant genus in oral microbiota and produced CH_3_SH, which is a strong contributor to halitosis. Suzuki et al. [35] reported that *Prevotella* might play a crucial role in providing amino acids during periodontitis. Another study reported a correlation between high H_2_S and CH_4_S levels and the growth of *Prevotella* [33]. In this study, *Rothia* and *Haemophilus* displayed significantly higher relative abundance in the control group than in the halitosis group. Seerangaiyan et al. [36] reported a positive correlation between *Prevotella* and oral malodor severity, which was contrary to the effect of *Haemophilus* and *Rothia*. *Streptococcus salivarius*, *Streptococcus milleri*, and *Streptococcus parasanguinis* have been found to be positively related to halitosis [37,38]. Bernardi et al. [39] reported that these microorganisms contributed significantly to IOH and could be regarded as treatment targets. Kazor et al. [9] reported that the species showing the strongest association with healthy subjects were *Streptococcus salivarius, Rothia mucilaginosa*, and an uncharacterized species of *Eubacterium*. Furthermore, based on an analysis of approximately 750 clones, the authors reported that the species associated most closely with halitosis were *Atopobium parvulum*, a phylotype of *Dialister*, *Eubacterium sulci*, a phylotype of the uncultivated phylum TM7, *Solobacterium moorei*, and a phylotype of *Streptococcus*.

### 2.3. Profiling of Saliva Metabolites

**Figure 3:**
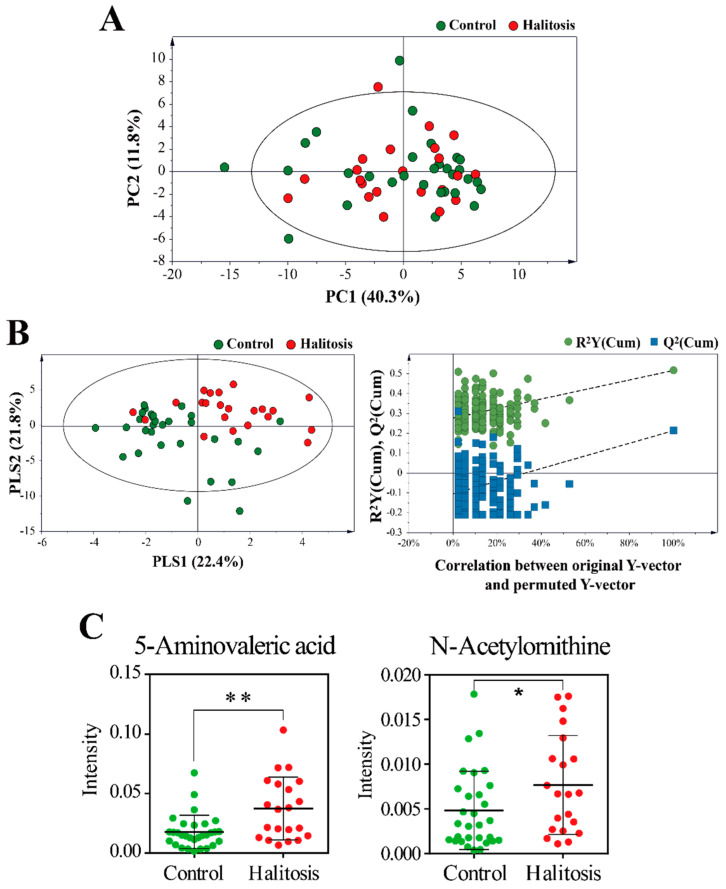
(**A**) The principal component analysis (PCA) built upon the gas chromatography–mass spectrometry (GC-MS) data of saliva samples from the control and the halitosis groups. (**B**) The supervised partial least-squares discriminant analysis (PLS-DA) show the discrimination between groups. The *R*^2^X, *R*^2^Y, and *Q*^2^ of PLS-DA are 0.442, 0.516, and 0.213, respectively. Permutation tests with 200 iterations were performed to validate the model. These tests compared the goodness of fit of the original model with the goodness of fit of randomly permuted models. (**C**) Scatter dot plots of two metabolites that contributed to the discrimination in the PLS-DA model (VIP > 1.0 and *p* < 0.05) between the control and halitosis groups. The y-axis represents the normalized intensity of each metabolite. * *p* < 0.05; ** *p* < 0.01.

The principal component analysis (PCA) was initially applied as an unsupervised statistical method to investigate the metabolic profile differences between the control and halitosis groups. However, the PCA model failed to confirm a clear separation between the two groups (Figure 3A). Partial least-squares discriminant analysis (PLS-DA) was then applied to further understand the different metabolite profiles and identify potential biomarkers (Figure 3B). The PLS-DA score plot revealed a diverse pattern between the control and halitosis groups, indicating that the metabolic profiles of saliva samples differed between the two groups. Permutation tests with 200 iterations were performed to assess whether or not the differences that classify the samples are significant. Through this test, *Q*^2^ and *R*^2^ values were found to be higher than their original values, proving the suitability and validity of this model.

Among the 66 metabolites identified in this study, variables significantly contributing to the discrimination between groups were selected based on a variable importance in the projection (VIP) > 1.0 and *p* < 0.05. Two potential biomarkers were identified, 5-aminovaleric acid and n-acetylornithine, both of which were found at significantly higher levels in the halitosis group (Figure 3C). Liebsch et al. [40] reported that 5-aminovaleric acid was associated with periodontitis, plaque, and calculus. Periodontitis is a major cause of halitosis [41]. Cadaverine, a foul-smelling diamine responsible for oral malodor, can be catabolized to 5-aminovalerate [42]. Ye et al. [43] reported a correlation between cadaverine levels in saliva and halitosis. *N*-acetylornithine can be converted to ornithine [44], which is subsequently converted to putrescine [45]. Putrescine is known to contribute to the putrid odor of conditions such as halitosis [46].

### 2.4. Correlation between Microbiome and Metabolome

**Figure 4:**
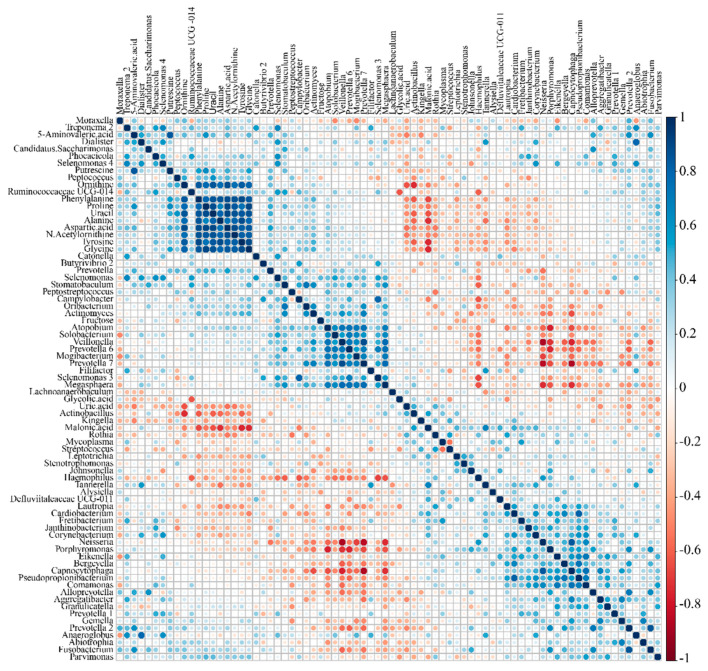
Heat map derived from correlation between the identified metabolites and microbiota in saliva samples.

To explore the relationships between the identified metabolites and microbiota, a heat map was generated using Spearman’s correlation coefficients (Figure 4). After correcting the multiple hypothesis test based on the false discovery rate (FDR) procedure, the analysis showed positive or negative Spearman’s correlations. In the current study, the relative abundances of *Prevotella*, *Alloprevotella*, and *Megasphaera* were significantly higher in the halitosis group (Figure 2) than in the control group. *Megasphaera* positively correlated with 5-aminovaleric acid (*r* = 0.185). Previous studies showed that *Prevotella* and *Alloprevotella* are commonly found in the halitosis group [11,47]. In the current study, *Alloprevotella* correlated positively with 5-aminovaleric acid (*r* = 0.51) and putrescine (*r* = 0.258). *Prevotella* correlated positively with n-acetylornithine (*r* = 0.341), ornithine (*r* = 0.423), putrescine (*r* = 0.423), and 5-aminovaleric acid (*r* = 0.481). A study reported that amines, including putrescine and cadaverine, caused halitosis [48]. Putrescine and cadaverine are produced from arginine and lysine [49,50] and have been associated with bacteria in dental plaque and severe periodontitis [11]. 5-aminovaleric acid participates in the cadaverine pathway [51,52], whereas n-acetylornithine, ornithine, and putrescine are associated with the putrescine pathway. These results suggested that *Prevotella* and *Alloprevotella* might be associated with putrescine and cadaverine production.

In our observations, the levels of 5-aminovaleric acid and n-acetylornithine were higher in the halitosis group than in the control group (Figure 3C). In the correlation between metabolites and bacteria, n-acetylornithine correlated positively with *Prevotella* (*r* = 0.350). 5-aminovaleric acid correlated positively with *Alloprevotella* (*r* = 0.513) and *Prevotella* (*r* = 0.482).

This study was conducted using only saliva samples. The results pertaining to some microorganisms and metabolites differ from those reported in previous studies using tongue samples. This discrepancy could be explained by a different microbiota composition between the tongue and saliva, as well as the limited number of research subjects and an unclear effect of halitosis on the patterns of microbiota or metabolites in saliva. We believe the main reason could be attributed to the difference between tongue and saliva samples, although the nature of such a difference remains to be determined. Nevertheless, because saliva collection is an accessible and user-friendly method of sampling, research based on saliva samples offers some advantages. Ling et al. [53] reported that the predominant microbiota in saliva was almost identical between children and adults, suggesting that salivary microorganisms could yield stable and reliable results. Therefore, future studies could address the exact effect of halitosis on microbiota and metabolites in saliva, as well as provide an accurate comparison with tongue samples.

## 3. Materials and Methods

### 3.1. Ethics Statement

The study was conducted in accordance with relevant guidelines and regulations and with the principles for human research. All participants provided written informed consent. The medical ethics committee at the Naju Korean Medicine Hospital of Dongshin University approved the study protocol (NJ-IRB-005). The study was conducted in accordance with the tenets of the Declaration of Helsinki (2013).

### 3.2. Halitosis Assessment

A total of 52 subjects were recruited from the Naju Korean Medicine Hospital of Dongshin University. All subjects were included in the study following careful halitosis examinations. Prior to their visit, subjects were instructed to (1) avoid consuming onions, garlic, and hot spices 48 h before the appointment; (2) refrain from alcohol intake and smoking 12 h prior to the halitosis examination; (3) abstain from normal oral hygiene procedures 3 h prior to the halitosis examination; and (4) avoid mint-containing products 3 h prior to the halitosis examination. Samples were collected between 08:00 and 10:00.

### 3.3. Exclusion Criteria

We excluded subjects with periodontitis or systemic diseases, smoker, pregnant women, those who used antimicrobial therapy and mouth rinses in the 3 months prior to the start of the study, those with a history of fever or cold in the previous 4 weeks, and those who failed to follow the instructions for the halitosis assessment.

### 3.4. Inclusion Criteria

To determine inclusion, the 52 participants were first assessed for VSC gases (H_2_S, CH_3_SH, and C_2_H_6_S) using OralChroma (Abilit Corporation, Osaka, Japan). A sample of breath air (5 mL) was taken by a syringe and injected into OralChroma. OralChroma VSC measurements were performed according to the manufacturer’s instructions. The halitosis group was selected based on H_2_S > 112 ppb, CH_3_SH > 26 ppb, and C_2_H_6_S > 8 ppb [54]. The control group had H_2_S < 112 ppb, CH_3_SH < 26 ppb, and C_2_H_6_S < 8 ppb.

### 3.5. Collection of Saliva

Unstimulated saliva collection was performed as recommended earlier [55,56]. Subjects were instructed to avoid brushing their teeth 3 h and eating 2 h prior to collecting a sample collection of saliva as well as to avoid swallowing for 2 min just before sampling. The total volume of saliva was collected in a plastic tube kept on ice, immediately labeled, transported to the laboratory in a portable iced container, and stored at −80 °C in a freezer. Frozen saliva was thawed on ice for microbial and metabolite analysis.

### 3.6. DNA Extraction and 16S rRNA Gene Amplicon Sequencing

For DNA extraction, 100 μL of saliva was extracted using the AccuFAST automation system (AccuGene, Incheon, Korea) in accordance with the manufacturer’s instructions. For MiSeq sequencing, bacterial genomic DNA amplification was performed using primers of 515 bp and 806 bp containing Nextera adaptor sequences and targeting the V4 hypervariable region of the 16S rRNA genes [57]. With KAPA HiFi HotStart ReadyMix, 16S rRNA genes were amplified in 25 polymerase chain reaction (PCR) (Roche, Pleasanton, CA, USA). The resulting PCR products (~250 bp) were purified using HiAccuBeads (AccuGene). Using MiSeq Reagent Kit v2 for 500 cycles (Illumina, San Diego, CA, USA), amplicon libraries at an equimolar ratio were pooled. The pooled libraries were sequenced using an Illumina MiSeq system. For the raw data sets, raw sequencing reads were subjected to reference-based chimeric filtering using VSEARCH v2.10.3 [58]. The chimeric filtered sequences were assigned to operational taxonomic units (OTUs) through OTU picking in the QIIME pipeline. The sequences were clustered using the UCLUST into OTUs based on the SILVA 132 (pre-clustered at 97% similarity threshold) database.

### 3.7. Sample Derivatization and GC–MS Analysis

After centrifuging the saliva sample at 13,000 rpm for 5 min at 4 °C, 100 μL of supernatant was pooled in a 1.5 mL Eppendorf tube. Then, 200 μL of cold methanol was added to precipitate protein. The mixture was then vortexed, centrifuged, and 100 µL of supernatant was freeze-dried. Following freeze-drying, 80 μL of O-methoxyamine hydrochloride (20 mg/mL) in pyridine solution was added to each freeze-dried saliva sample. The samples were then vortex-mixed for 30 s and incubated at 30 °C for 90 min in the dark. Approximately 30 μL of *N*-methyl-*N*-trimethylsilyl-trifluoroacetamide with 1% trimethylchlorosilane was added to each sample for the silylation process, followed by vortexing for 30 s and incubation at 37 °C for 30 min. Approximately 10 μL of ribitol (0.5 mg/L) was used as an internal standard.

The derivatized samples were analyzed using GC-MS (QP2020, Shimadzu, Kyoto, Japan). An Rtx-5MS fused silica capillary column (30 m × 0.25 mm, 0.25 µm; J&W Scientific, Folsom, CA, USA) was used for the separation of metabolites. The front inlet temperature was set to 230 °C. The column temperature was maintained at 80 °C for 2 min isothermally, then raised by 15 °C/min to 330 °C, and held there for 6 min isothermally. The transfer line and ion source temperatures were 250 °C and 200 °C, respectively. Using a 70 eV electron beam, ionization was achieved. The helium gas flow rate through the column was 1 mL/min. Approximately 20 scans/s were recorded in a mass range of 85–500 *m*/*z*. A GC solution (Shimadzu, Kyoto, Japan) was employed to obtain chromatograms and mass spectra. The stability and performance of the instrument were measured along with the reproducibility of the sample treatment procedure. Quality control was assessed every five samples during the run.

### 3.8. Data Processing and Multivariate Analysis

The GC-MS data were converted to a netCDF format file and processed using MetAlign software for peak detection and alignment [59]. MetAlign parameters were set according to the AIoutput scaling requirements: a peak slope factor of 2, peak threshold of 10, average peak width at half height of 25, and peak threshold factor of 4. These settings corresponded to a retention time of 3–26 min and mass range of 85–500 *m*/*z*. The result of the data (CSV) was imported into AIoutput software for peak prediction and identification [60]. After feature intensities were normalized relative to the intensity of the internal standard (retention time 11.205 min, *m*/*z* 147), multivariate statistical analyses were performed. To visualize the variance of metabolites, PCA and PLS-DA of GC-MS data were performed using SIMCA-P, version 15.0 (Umetrics, Umea, Sweden). For model validation, a 200-fold cross validation was performed. Metabolites with a VIP score greater than 1.0 and *p*-value < 0.05 using the Student’s *t*-test were considered to have discriminatory power to distinguish between the two groups. Multiple testing was corrected, using the positive FDR (type 1 error) by computing the *q*-values after the *t*-test. Metabolites were identified by comparing their mass spectra with the AIoutput software, NIST library, and the human metabolome database (HMDB).

### 3.9. Correlation Analysis

The associations between the metabolites and microorganisms in saliva samples were assessed using the Spearman’s rank correlation analysis. The FDR of 5% was applied to all tests to correct for multiple testing.

## 4. Conclusions

Halitosis is caused by metabolites produced by oral microorganisms. Therefore, it is important to clarify the correlation between oral microbes and metabolites. This study helps in exploring the mechanism underlying halitosis by identifying the microbiota and metabolites correlated with this condition.

## Figures and Tables

**Table 1:** Demographic and clinical characteristics of the study subjects.

Clinical Parameters		Control (*n* = 30)	Intra-Oral Halitosis (*n* = 22)
Age (years)		38.50 ± 11.94	43.43 ± 15.73
Sex	Female	20	13
	Male	10	9
H_2_S ^1^		27.50 ± 25.81	806.77 ± 866.08 *** ^1^
CH_3_SH		7.10 ± 6.14	213.41 ± 217.14 ***
(CH_3_)_2_S		39.73 ± 49.74	82.77 ± 71.20 *

^1^ The levels of H_2_S, CH_3_SH, and (CH_3_)_2_S were measured in parts per billion (ppb). Continuous variables are represented as mean ± standard deviation. Symbols (*) indicate significant difference (* *p* < 0.05; *** *p* < 0.001).

## Data Availability

The data presented in this study are available on request from the corresponding author.
